# Supplementary material for: Effects of Cerebrospinal Fluids from Alzheimer and Non-Alzheimer Patients on Neurons–Astrocytes–Microglia Co-Culture
Source: Int J Mol Sci. 2024 Feb 21;25(5):2510. doi: 10.3390/ijms25052510 (PMC10931816; doi:10.3390/ijms25052510)
Supplement: Supplementary file 1 [file ijms-25-02510-s001.zip › ijms-2844799-supplementary.pdf]

Supplementary:

Figure S1 – Gelatin zymographies of Conditionated Media (CM) of NAM co-cultures.
(A'-1, A'-2, A'-3, and A'-4) and (A"-1, A"-2, A"-3, and A"-4) zymographies of CM of NAM treated with CSF from AD subjects at 24 and 48 hrs, (B-1, B-2, B-3, and B-4) zymographies of CM of NAM treated with CSF from non AD subjects at 24 and 48 hrs.
In (**a**) developed in presence of 2 mmol/L $CaCl_2$, Tris-HCl buffer (50 mmol/L; pH 7.4) containing 1.5% Triton X-100 and 0.02% Na_3 Azide and used in evaluation by integrated density grey-color, Image J64 program.
In (**b**) developed in presence of Tris-HCl buffer (50 mmol/L; pH 7.4), containing 1.5% Triton X-100 and 0.02% Na_3 Azide plus 2 mmol/L EDTA.

Figure S2 – Gelatin zymographies of Extracts (Ext) from NAM co-cultures.
(A'-1, A'-2, A'-3, and A'-4) and (A"-1, A"-2, A"-3, and A"-4) zymographies of Ext from NAM treated with CSF from AD subjects at 24 and 48 hrs, (B-1, B-2, B-3, and B-4) zymographies of Ext from NAM treated with CSF from non AD subjects at 24 and 48 hrs.
In (**a**) developed in presence of 2 mmol/L $CaCl_2$, Tris-HCl buffer (50 mmol/L; pH 7.4) containing 1.5% Triton X-100 and 0.02% Na_3 Azide and used in evaluation by integrated density grey-color, Image J64 program.
In (**b**) developed in presence of Tris-HCl buffer (50 mmol/L; pH 7.4), containing 1.5% Triton X-100 and 0.02% Na_3 Azide plus 2 mmol/L EDTA and used in evaluation by integrated density grey-color, Image J64 program.

Figure S3 – Gelatin zymographies CM of CSF from different patients.
(A'-1, A'-2, and A'-3) and (A"-2, and A"-4) CM zymographies of CSF from AD subjects at 24 hrs, (B-4) CM zymographies of CSF from NAM treated with CSF from non AD subject at 24 hrs.

Figure S1

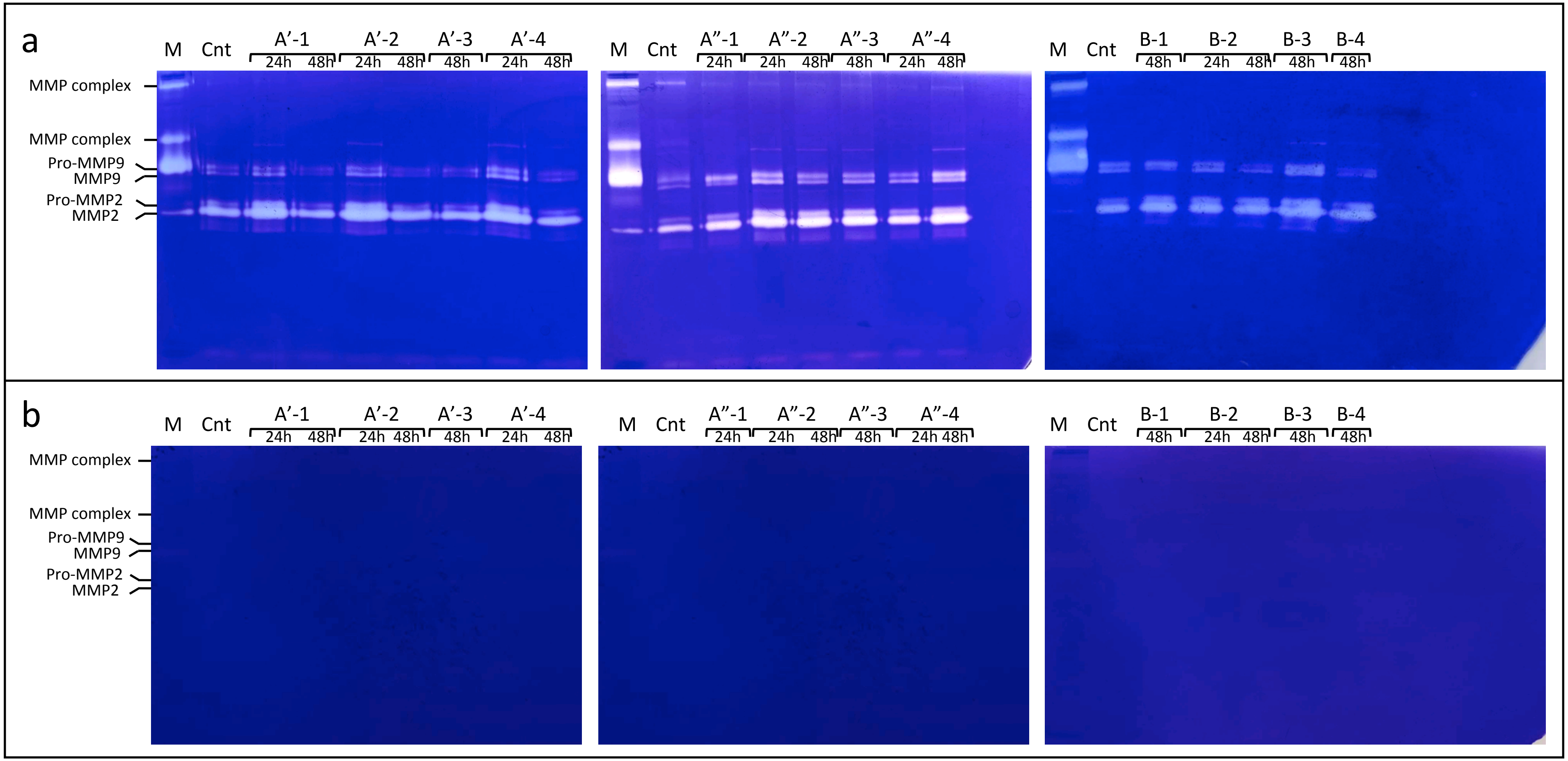

a
b
M
Cnt
A'-1
A'-2
A'-3
A'-4
A"-1
A"-2
A"-3
A"-4
B-1
B-2
B-3
B-4
24h
48h
MMP complex
MMP complex
Pro-MMP9
MMP9
Pro-MMP2
MMP2

Figure S2

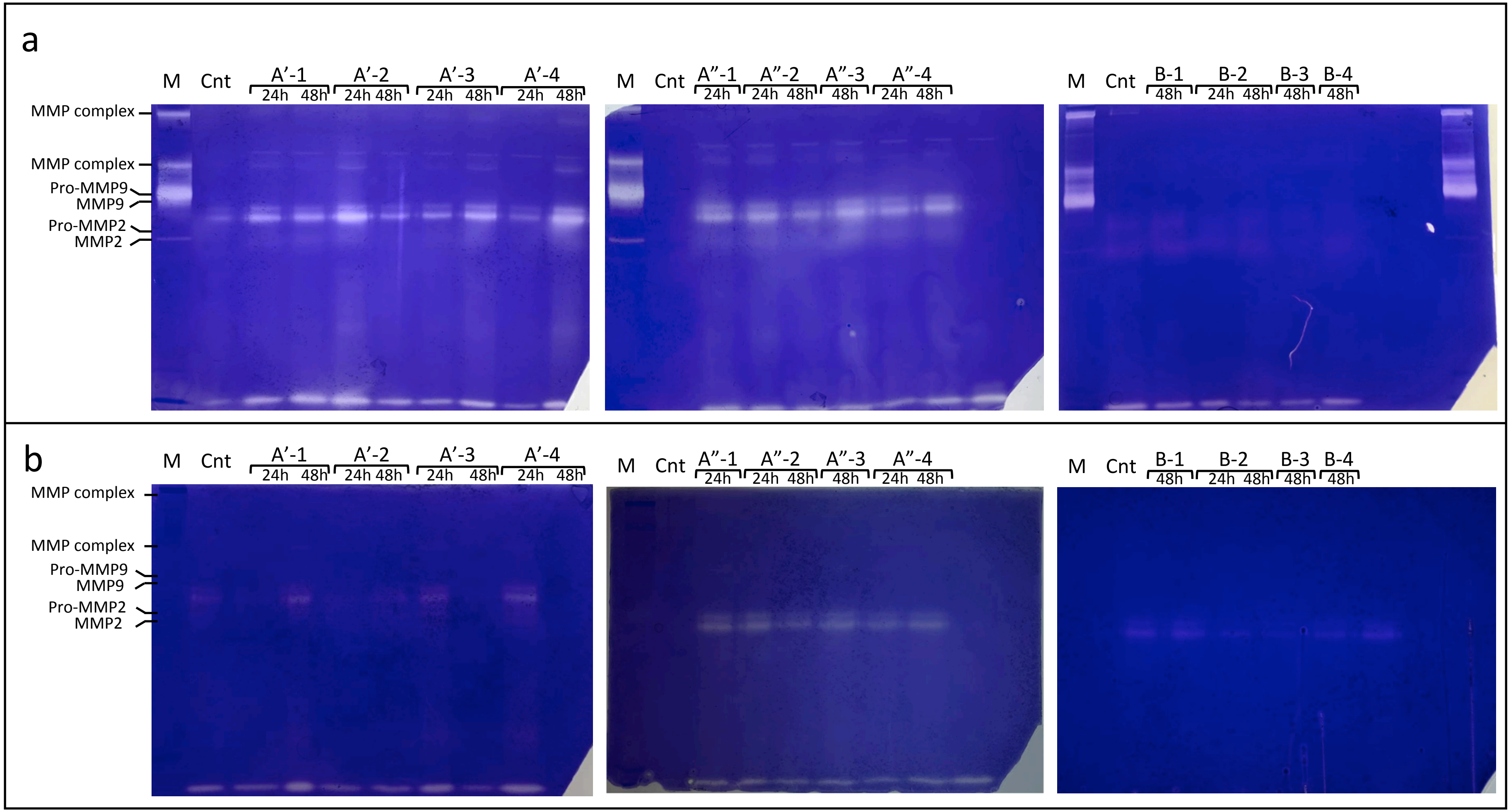

a
M
Cnt
A’-1
A’-2
A’-3
A’-4
24h
48h
MMP complex
MMP complex
Pro-MMP9
MMP9
Pro-MMP2
MMP2
A”-1
A”-2
A”-3
A”-4
B-1
B-2
B-3
B-4
b

Figure S3

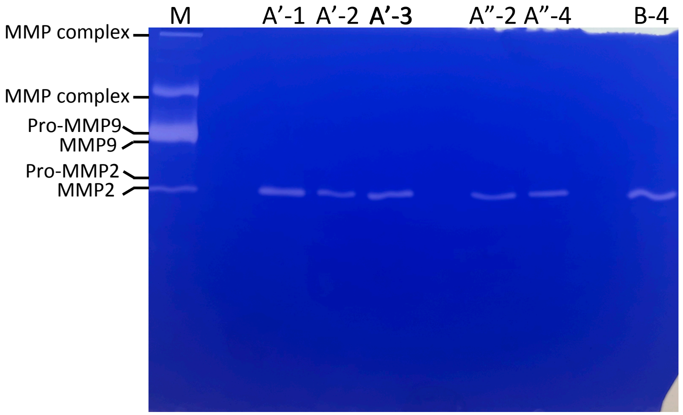

M
A'-1
A'-2
A'-3
A"-2
A"-4
B-4
MMP complex
MMP complex
Pro-MMP9
MMP9
Pro-MMP2
MMP2
